# Interaction of CD99 and its ligands regulates immunoregulatory pathways in NK cells

**DOI:** 10.3389/fimmu.2026.1840402

**Published:** 2026-06-04

**Authors:** Myint Myat Thu, Nuchjira Takheaw, Witida Laopajon, Watchara Kasinrerk, Supansa Pata

**Affiliations:** 1 Division of Clinical Immunology, Department of Medical Technology, Faculty of Associated Medical Sciences, Chiang Mai University, Chiang Mai, Thailand; 2 Biomedical Technology Research Center, Faculty of Associated Medical Sciences, Chiang Mai University, Chiang Mai, Thailand

**Keywords:** activating receptors, CD99HIgG, co-stimulatory receptors, natural cytotoxicity receptors (NCRs), natural killer (NK) cells

## Abstract

Natural killer (NK) cells play a key role in immune defense against tumors and viral infections through a balance of activating and inhibitory receptors. CD99 is a transmembrane glycoprotein involved in many biological processes; however, its role in NK cell receptor modulation remains incompletely understood. In this study, we investigated the effect of CD99 engagement on NK cells using a recombinant CD99 fusion protein upon stimulation with cytokine, interleukin (IL)-2. Peripheral blood mononuclear cells (PBMCs) and purified NK cells were analyzed to evaluate changes in several receptor expressions. Flow cytometric analysis demonstrated that the interaction between CD99 and its ligands modulates NK cell phenotypes, particularly through downregulation of natural cytotoxicity receptors (NCRs). Differential effects were observed between PBMCs and purified NK cell cultures, indicating the surrounding immune cell environment influences CD99-mediated immunomodulatory responses. Despite receptor modulation, no significant changes in cytokine production were observed in NK cells. These findings suggest that CD99 and its ligand interactions contribute to the regulation of NK cell activation states and receptor dynamics under activated conditions in a context-dependent manner.

## Introduction

NK cells are innate lymphoid cells that play a crucial role in the immune defense against cancers and viral infections. Their activity depends on the balance between activating and inhibitory receptors, which are modulated by cytokines, metabolic signals, and tissue ligands (1). Activating receptors such as DNAM-1, NKG2D, and CD16 contribute to NK cell recognition and subsequent functional responses against malignant or infected cells (2–4). Among activating receptors, NCRs including NKp30, NKp44 and NKp46 represent a major receptor family essential for the surveillance of tumor-derived and viral ligands (5). In addition, activation-associated and co-stimulatory markers, including CD25, CD69, CD27, and CD137 reflect NK cell activation and functional responsiveness (6, 7). Disruption of this finely tuned receptor balance can compromise NK cell-mediated immune surveillance, leading to immune evasion, persistent inflammation, and reduced antitumor responses. The concept of immune checkpoint regulation, initially applied to T cells and subsequently extended to NK cells, illustrates that NK cell dysfunction in cancer is frequently attributable to receptor plasticity rather than solely a deficiency in cytotoxic activity (8, 9). Cytokines, including IL-2, IL-15, IL-6, TNF-α, and TGF-β, regulate NK cell activity, influencing receptor expression, signaling pathways, metabolic processes, and cellular responsiveness (9, 10). Consequently, these effects lead to phenotypic modifications and states analogous to exhaustion. In tumor and inflamed environments, cytokine conditioning reprograms NK cells and modifies their receptor profiles (8, 11–14). Recent scholarly attention has transitioned to alternative immunoregulatory molecules that modulate NK cell activation.

CD99 is a broadly expressed transmembrane glycoprotein involved in leukocyte adhesion, transendothelial migration, immune synapse formation, and lymphocyte activation (15, 16). Recent pan-cancer analyses further demonstrate that CD99 expression correlates with immune infiltration patterns and immune checkpoint signatures across multiple tumor types, suggesting that CD99 participates in shaping immune microenvironments (17). Furthermore, interactions between CD99 and its ligands have been identified within innate immune compartments, including monocytes, NK cells, and dendritic cells (18, 19). CD99 engagement has demonstrated the capacity to modulate inflammatory cytokines such as IL-6 and TNF-α across immune environments (18, 20). Nevertheless, the implications of CD99 engagement on NK receptor architecture, particularly NCR-associated phenotypic heterogeneity and activation, remain only partially understood.

This study employed a recombinant CD99 fusion protein (CD99HIgG) (18, 20) to investigate CD99 ligand interactions in NK cells, focusing on how engagement modulates activating receptors, NCRs, and co-stimulatory receptor expression during IL-2 activation. By analyzing both PBMCs and purified NK cells, the study aims to clarify the role of CD99 in regulating NK cell activation and phenotypic diversity. We demonstrated that the interaction between CD99 and its ligands modulates NK cell phenotypes by downregulating NCR expression and altering the distribution of NCR-associated NK cell populations, without significantly affecting cytokine production. These findings suggest a previously unrecognized role of CD99-CD99 ligand interaction in shaping NK cell activation states and phenotypic heterogeneity.

## Materials and methods

### Antibodies and reagents

PECF594-conjugated anti-CD3 mAb (Cat# 562280) was obtained from BD Biosciences (San Jose, CA, USA). PECy5-conjugated anti-CD56 mAb (Cat# 318308), FITC-conjugated anti-CD14 mAb (Cat# 301804), FITC-conjugated anti-CD19 mAb (Cat# 302206), Brilliant Violet (BV) 421-conjugated anti-NKp30 mAb (Cat# 325228), BV510-conjugated anti-NKp46 mAb (Cat# 331924), BV711-conjugated anti-CD16 mAb (Cat# 302044), FITC-conjugated anti-DNAM-1 mAb (Cat# 338304), PE-conjugated anti-NKG2D mAb (Cat# 320806), PECy7-conjugated anti-NKp44 mAb (Cat# 325116), FITC-conjugated anti-CD25 mAb (Cat# 356106), PE-conjugated anti-CD27 mAb (Cat# 302808), BV421-conjugated anti-CD69 mAb (Cat# 310930), PECy7-conjugated anti-CD137 mAb (Cat# 309818), PE-conjugated anti-IL6 mAb (Cat# 501107), PECy7-conjugated anti-TNF-α mAb (Cat# 502909), BV421-conjugated anti-IFN-γ mAb (Cat# 502532), PE-conjugated IgG mAb (Cat# 400140), PECy7-conjugated IgG mAb (Cat# 400126), and BV421-conjugated IgG mAb (Cat# 400158) were purchased from BioLegend (San Diego, CA, USA). NK isolation kit and LS separation column were obtained from Miltenyi Biotech (BergischGladbach, Germany). Human IgG (HIgG) was purified from AB serum donor. Recombinant CD99HIgG was produced in-house as previously described (18, 20). PBMCs were prepared from residual peripheral blood samples collected from anonymous donors.

### Cell preparation (PBMCs and purified NK cells)

PBMCs were prepared by Ficoll-Hypaque density gradient centrifugation. NK cells were purified from PBMCs by using the NK cell isolation kit and LS separation column according to the manufacturer’s protocol. After isolation, the purity of purified NK cells was > 90%. The cell concentration was adjusted to 2×10^6^ cells/ml for PBMCs and 5×10^6^ cells/ml for purified NK cells to stimulate with IL-2.

### Effect of CD99 and CD99 ligand interaction on NK cell receptor expression

PBMCs and purified NK cells were stimulated with 250 IU/ml of IL-2 in the presence or absence of recombinant CD99HIgG or control HIgG (10 ug/ml). The cells were incubated at 37 °C in 5% CO_2_ incubator for 1 and 3 days. After incubation, cells were stained with two cocktail antibodies to determine the expression of activating receptors (DNAM-1, CD16, NKG2D), NCRs (NKp30, NKp44, NKp46), activation associated receptors (CD25, CD69) and co-stimulatory receptors (CD27, CD137) on NK cells. The first cocktail antibodies include BV421-conjugated anti-NKp30 mAb, BV510-conjugated anti-NKp46 mAb, BV711-conjugated anti-CD16 mAb, FITC-conjugated anti-DNAM-1 mAb, PE-conjugated anti-NKG2D mAb, and PECy7-conjugated anti-NKp44 mAb. The second cocktail antibodies consist of and co-stimulatory and activation-associated molecules, FITC-conjugated anti-CD25 mAb, PE-conjugated anti-CD27 mAb, BV421-conjugated anti-CD69 mAb, and PECy7-conjugated anti-CD137 mAb. PE-conjugated mouse IgG mAb, PECy7-conjugated mouse IgG mAb, and BV421-conjugated mouse IgG mAb were used as isotype-matched control mAbs. NK cells from PBMCs were identified by staining with PECF594-conjugated CD3 mAb and PECy5-conjugated CD56 mAb. After staining, molecular expression was analyzed by flow cytometry (BD FACSCelesta™) using FlowJo software v.10.10.0.

### Flow cytometry analysis

The linear expression profiles of the tested molecules were analyzed by FlowJo software using manual gating analysis. The expression levels of each tested molecule were evaluated based on geometric mean fluorescence intensity (GeoMFI) and the percentage of marker positive cells using Comparison Population tool in FlowJo to minimize user-dependent variability associated with manual gating. For each cocktail staining, the percentage of marker positive cells was determined by comparison with the corresponding isotype-matched staining control. The Overton % Positive values were used to calculate percentage positivity for subsequent statistical analysis. A representative analysis using the Comparison Population tool is shown in **Supplementary Figure 1**.

**Figure 1:**
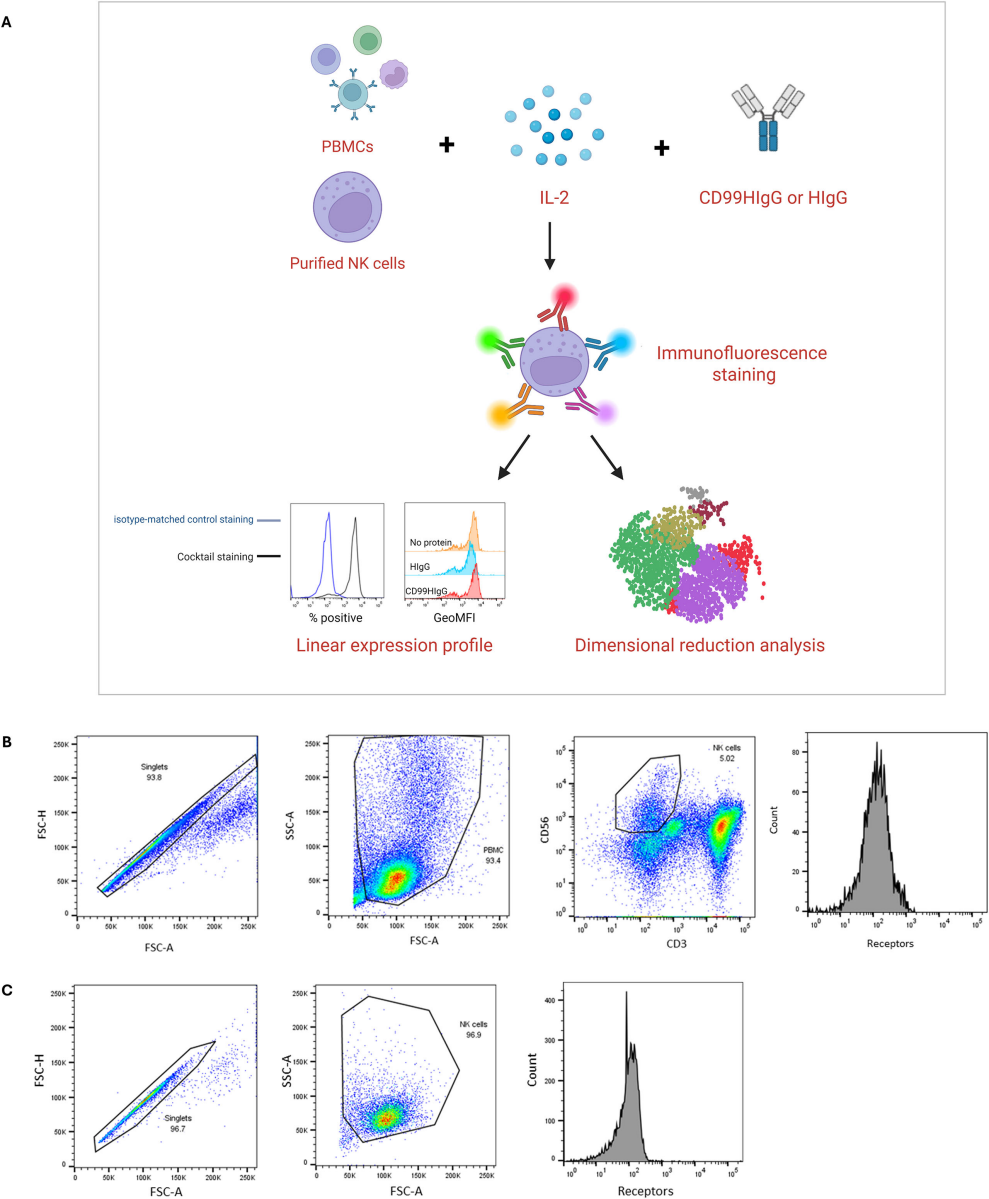
Experimental design and flow cytometry gating strategy for assessing CD99- CD99 ligand modulation of NK cell receptor expression. **(A)** Schematic overview of the experimental workflow. PBMCs and purified NK cells were stimulated with IL-2 in the presence of recombinant CD99HIgG or control HIgG. Receptor expression was evaluated at two time points: 1 day after cytokine stimulation (D1) and after 3 days (D3). Created in BioRender. Pata, S (2026). https://BioRender.com/bxz951p. **(B)** Gating strategy used for identification of NK cells within PBMC cultures. Singlets were initially gated using FSC-A and FSC-H, followed by selection of the PBMC population based on FSC-A and SSC-A. From PBMC population, CD3^-^CD56^+^ NK cells were gated and subsequently analyzed the receptor expression. **(C)** Gating strategy for purified NK cell cultures used to assess receptor modulation following CD99HIgG exposure. Single cells were initially gated using FSC-A and FSC-H. Purified NK cells were identified based on FSC-A and SSC-A followed by analysis of receptor expression within the purified NK cell population.

The non-linear expression profiles were visualized using FlowJo Plugins, including Uniform Manifold Approximation and Projection (UMAP), Flow Self-Organizing Map (FlowSOM), and Cluster Explorer. CD3^-^CD56^+^ NK cells gated from whole PBMCs (**Figure 1B**) and used for UMAP analysis. Dimensionality reduction was performed using DNAM-1, CD16, NKG2D, NKp30, NKp44, and NKp46 to visualize phenotypically distinct cell populations. A total of 10 clusters on the resulting UMAP plot were identified by FlowSOM based on the same markers used for UMAP analysis. Cluster Explorer was subsequently used following dimensionality reduction and clustering to generate overlaid dimensionality reduction plots, profile charts and heatmaps. Based on the relative expression intensity observed in Cluster Explorer, the expression levels of NCRs within each cluster were comparatively ranked as high (+3), moderate (+2), low (+1) and negative (–). Clusters were then classified as NCR High, NCR Medium and NCR Low according to the overall expression patterns of NCR-associated markers. Clusters predominantly showing +3 and +2 expression levels for these markers were classified as NCR High, clusters mainly showing +2 and +1 expression levels were classified as NCR Medium, and clusters mainly showing +1 or negative expression levels were classified as NCR Low. This classification was based on relative comparison across all identified clusters rather than using fixed numerical thresholds. The relative abundance of these clusters was subsequently compared among CD99HIgG, HIgG and No protein treated groups.

### Effect of CD99 and CD99 ligand interaction in cytokine production upon stimulation with IL-2

PBMCs were cultured overnight with 250 IU/ml of IL-2 and subsequently treated with or without CD99HIgG or control HIgG (10 ug/ml). After cultivation for 1 h at 37 °C in a 5% CO_2_ incubator, protein transport inhibitors (1 μg/ml brefeldin A and 1 μM monensin) were added and continuously incubated at 37 °C in a 5% CO_2_ incubator for 5 h. Cells were surface stained with cocktail antibodies for analysis of different immune cell populations (NK cells, monocytes and CD3^+^ cells) using PECF594-conjugated anti CD3 mAb, PECy5-conjugated anti-CD56 mAb, FITC-conjugated anti-CD14 and FITC-conjugated anti CD19 mAbs, respectively. Intracellular staining was performed using 4% paraformaldehyde and 0.1% saponin, followed by staining with PE-conjugated anti-IL-6 mAb, PECy7-conjugated anti-TNF-α mAb, BV421-conjuated anti-IFN-γ mAb. PE-conjugated mouse IgG mAb, PECy7-conjugated mouse IgG mAb and BV421-conjugated mouse IgG mAb were used as isotype-matched control mAbs. After staining, cytokine production was examined by flow cytometry (BD FACSCelesta™) using FlowJo software v.10.10.0. Cytokine production was determined based on GeoMFI and percent positive cells using the Comparison Population tool in FlowJo by comparing tested staining with corresponding isotype-matched control staining. Percentage positivity was calculated using the Overton % Positive for subsequent statistical analysis.

### Statistical analysis

Descriptive data were presented in mean ± SD as indicated in each experiment. Statistical analysis was carried out using GraphPad Prism 10 software (San Diego, CA, USA). Comparisons between matched donor samples (CD99HIgG and HIgG) were analyzed using paired t test, while comparisons among three treatment conditions were analyzed using repeated-measures one-way ANOVA with Tukey’s multiple comparisons. Statistical significance was set at p < 0.05.

## Results

### Experimental design

To study the effect of CD99-CD99 ligand interaction in NK cell receptor expression, PBMCs and purified NK cells were stimulated with IL-2 in the presence of CD99HIgG or control protein, HIgG (**Figure 1A**). The changes in NK cell receptors were studied at different times: 1 day after the initial stimulation with cytokines (Day 1; D1), and after 3 days of culture (Day 3; D3).

The treated cells were assayed for activating receptors, NCRs, and co-stimulatory molecules by direct immunofluorescence staining. The expression profiles of the tested molecules were analyzed by FlowJo with manual two-dimensional analysis based on GeoMFI and percent positive. The non-linear expression profiles were visualized with UMAP, FlowSOM, and Cluster Explorer. The gating strategy used for identifying NK cells in PBMCs and analyzing expression of tested molecules was shown in **Figure 1B**. The gating strategy used to investigate the expression of tested molecules in purified NK cells was shown in **Figure 1C**.

### CD99-CD99 ligand interaction alters activating receptors and NCR expression in NK cells

**Figure 2:**
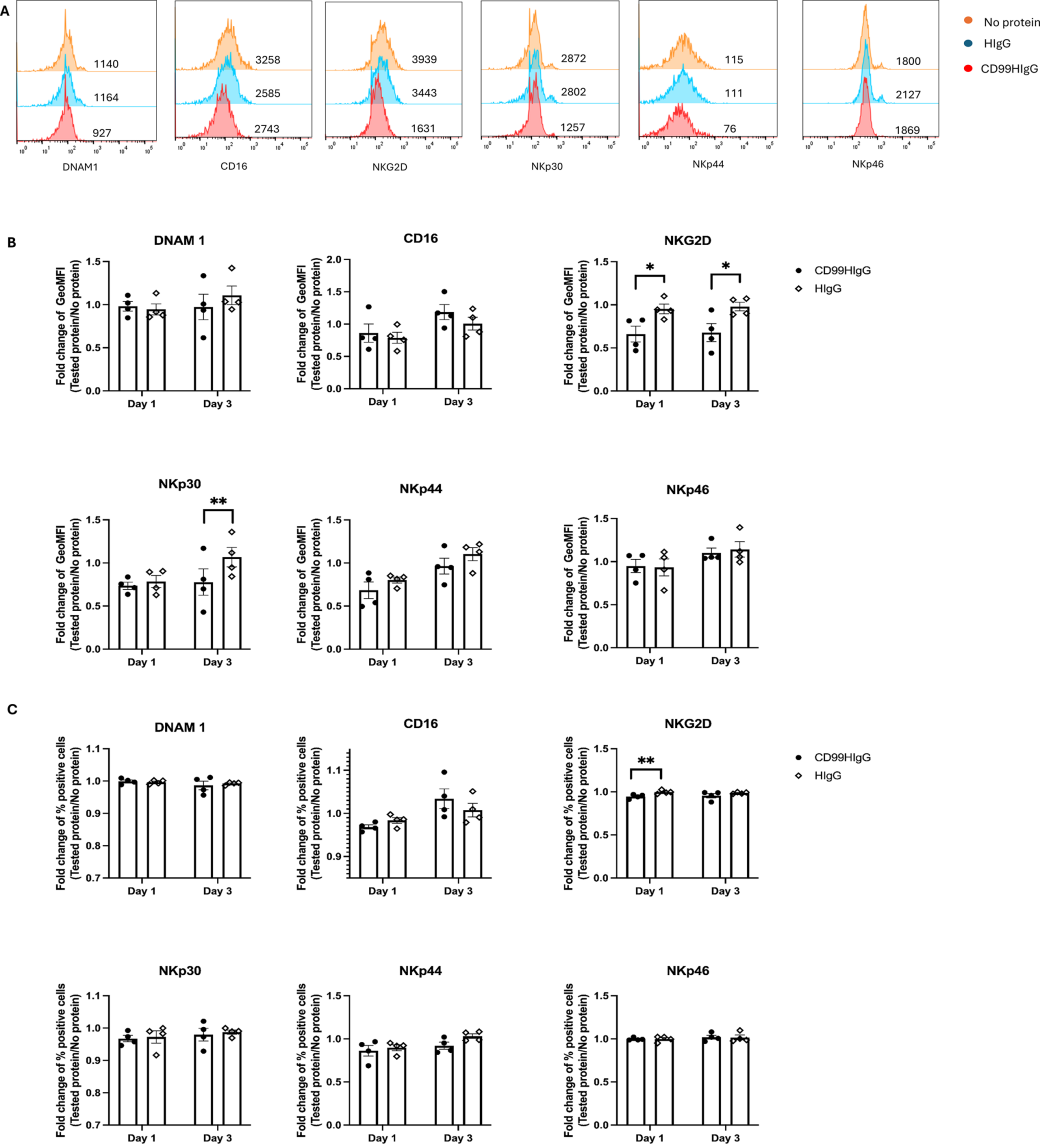
CD99-ligand interaction alters NK cell activation phenotypes under IL-2 stimulation. PBMCs were stimulated with IL-2 in the presence of CD99HIgG or control, HIgG. **(A)** Representative flow cytometry plots of D3 showing the GeoMFI of activating receptors and NCRs under each treatment condition. **(B)** Fold changes in the GeoMFI of activating receptors and NCRs on NK cells within PBMCs. **(C)** Fold changes in the percentage of receptor positive NK cells within PBMCs. Bar graphs presented as mean ± SD (n=4). Statistical analysis was performed using a paired t-test assuming normal distribution. *p < 0.05, **p < 0.01.

We first assessed the expression of activating receptors (DNAM-1, CD16, NKG2D) and NCRs (NKp30, NKp44, NKp46) in NK cell subsets from PBMCs. Based on GeoMFI, most tested receptors were downregulated in the presence of CD99HIgG, but not in the HIgG protein control, particularly on D3 (**Figures 2A, B**). Representative flow cytometry (FACS) profiles showing GeoMFI of each receptor under different treatment conditions are presented in **Figure 2A**. Among these receptors, the GeoMFI of NKG2D was significantly decreased on both D1 and D3 (p = 0.0211 and p = 0.0193) compared with HIgG control, whereas one of NCRs, NKp30 showed significant downregulation only in D3 (p = 0.0089). However, the remaining receptors showed no statistically significant differences between CD99HIgG and the control HIgG (**Figure 2B**). Subsequently, we analyzed the proportion of receptor positive NK cells following IL-2 stimulation. CD99HIgG treatment significantly reduced the frequencies of NKG2D^+^ NK cells on D1 in comparison with control HIgG (p = 0.0057), as illustrated in **Figure 2C**. The percent positive of remaining receptors did not show any significant difference.

### CD99-CD99 ligand interaction shifts NK cells toward NCR-low phenotypes, shown by high-dimensional analysis

**Figure 3:**
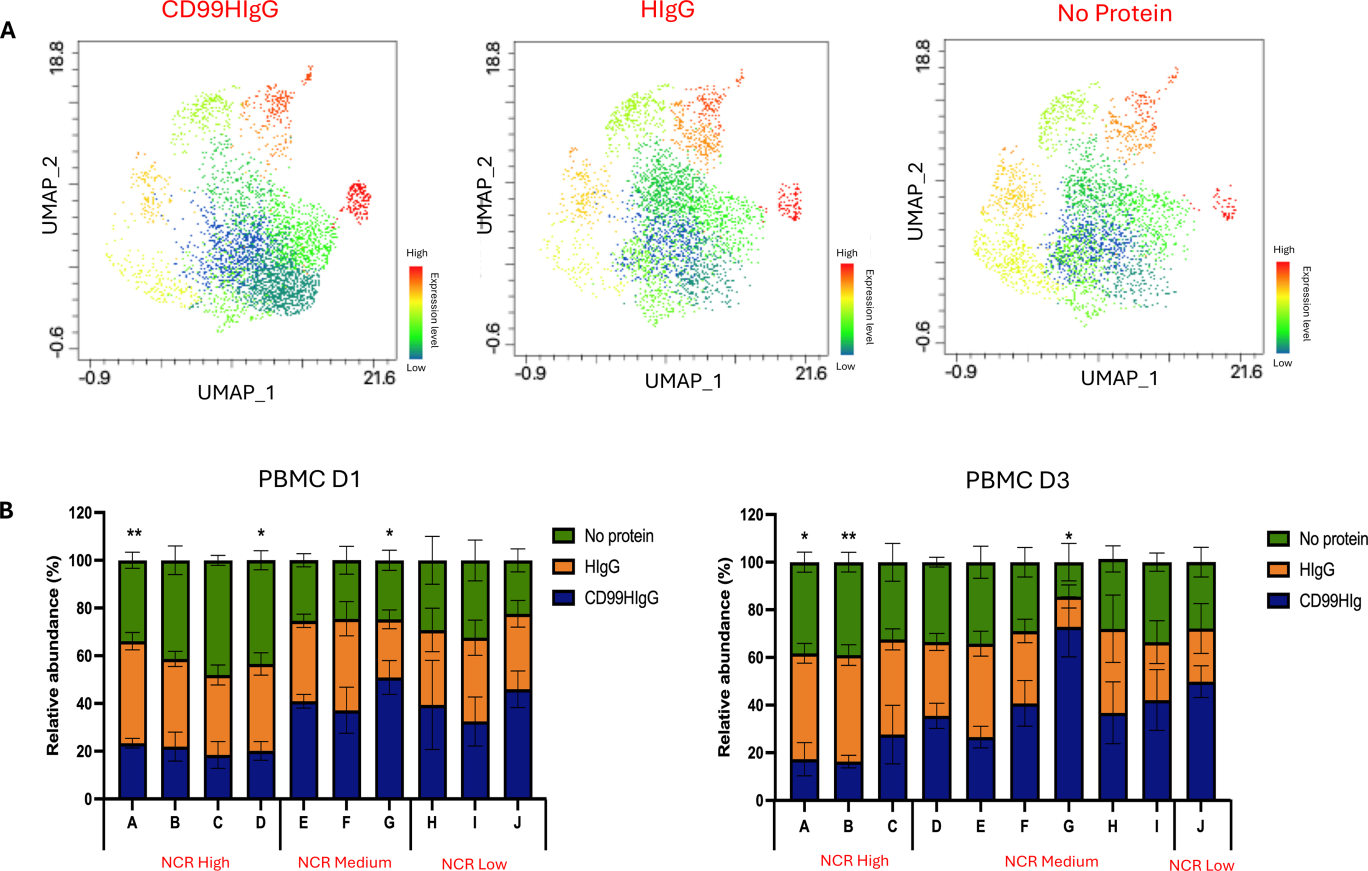
CD99HIgG alters the distribution of NCR-defined NK cell phenotypes under IL-2 stimulation. **(A)** Representative UMAP visualization of NK cell populations generated using NCRs and activation markers. Each dot represents a single cell, and the color scale indicates receptor expression level (low to high). Comparisons among CD99HIgG, HIgG control, and the no-protein condition are shown. **(B)** Stacked bar graphs showing the relative abundance (%) of NK cell clusters grouped by NCR expression levels (NCR High, NCR Medium, and NCR Low) (n=4) under different treatment conditions at PBMC D1 and PBMC D3. Colors represent treatment groups: CD99HIgG (blue), HIgG (orange), and No protein (green). NK cell clusters (A–J) were defined based on receptor expression patterns identified by FlowSOM and Cluster Explorer analysis. Statistical analysis was performed using repeated-measures one-way ANOVA with Tukey’s multiple comparisons and assuming normal distribution comparing among CD99HIgG, HIgG and No protein conditions. Statistical significance was shown the comparison between CD99HIgG and HIgG. *p < 0.05, **p < 0.01.

To investigate whether CD99-CD99 ligand interaction influences overall NK cell phenotypic composition, high-dimensional analysis was employed. Dimensionality reduction UMAP visualization revealed distinct NK cell populations based on NCRs and activation marker (DNAM-1, CD16, and NKG2D) expression levels across treatment conditions (**Figure 3A**). Although the overall cellular distribution remained largely similar among conditions, differences in the distribution of NK cell clusters were observed in the presence of CD99HIgG compared with the control groups (**Figure 3A**).

To further quantify these differences, NK cell clusters were grouped into NCR High, NCR Medium, and NCR Low subsets, and their relative abundance was analyzed at D1 and D3 of PBMC culture (**Figure 3B**). In both D1 and D3, there was significantly reduced of two NCR High clusters in the presence of CD99HIgG compared to control HIgG. Consistent with the conventional flow cytometry results showing reduced NCR expression, cluster-based analysis revealed a reduced abundance of NCR High NK cell phenotypes in the presence of CD99HIgG. When NK cell clusters were grouped into NCR High, NCR Medium, and NCR Low subsets, CD99HIgG treatment shifted the distribution toward NCR Medium and NCR Low populations at both D1 and D3 (**Figure 3B**). Together, these findings indicate that CD99– CD99 ligand interaction modulates NK cell phenotypes by downregulating NCR expression and altering the distribution of NCR defined NK cell subsets during IL-2 stimulation.

### CD99-CD99 ligand interaction influences NK cell co-stimulatory molecule expression

**Figure 4:**
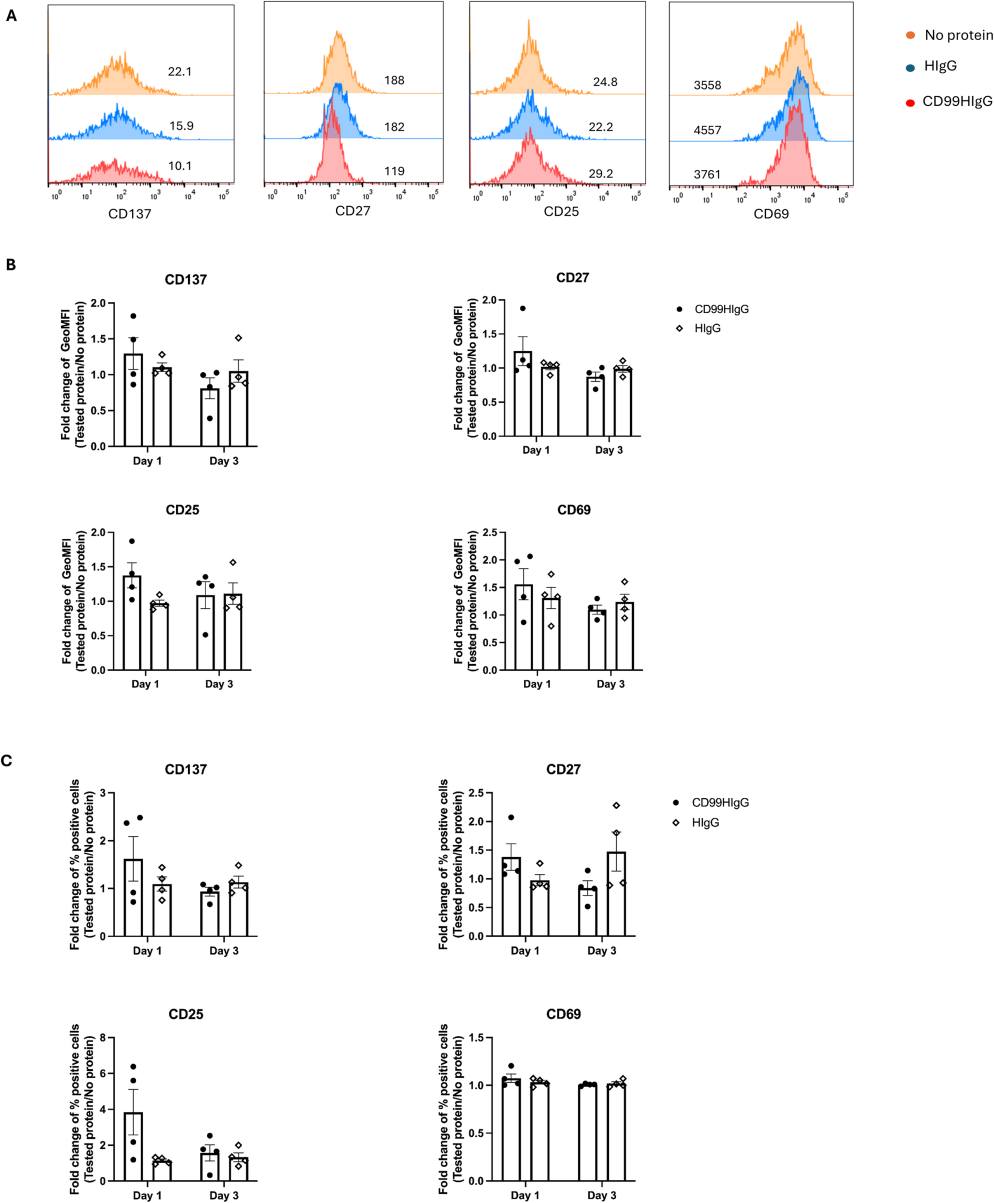
CD99- CD99 ligand interaction alters NK cell co-stimulatory and activation-associated receptor expression under IL-2 stimulation. PBMCs were stimulated with IL-2 in the presence of CD99HIgG or control HIgG. **(A)** Representative flow cytometry histograms of D3 after stimulation comparing GeoMFI of activation-associated and co-stimulatory receptors under each treatment condition. **(B)** Fold changes in the GeoMFI of activation-associated and co-stimulatory receptors on NK cells within PBMCs. **(C)** Fold changes in the percentage of receptor positive NK cells within PBMCs. Bar graphs presented as mean ± SD (n=4). Statistical analysis was performed using a paired t-test assuming normal distribution.

Upon IL-2 stimulation, the GeoMFI of co-stimulatory and activation-associated molecules (CD27, CD137, CD69, and CD25) was analyzed in NK cell subsets within PBMCs under different treatment conditions. Representative FACS profiles illustrating these changes are shown (**Figure 4A**). Although, all tested molecules tended to increase in the presence of CD99HIgG at D1, no statistically significant differences were observed in either GeoMFI or percentage of positive cells at D1 and D3 (**Figures 4B**, **C**).

### Direct effects of CD99-CD99 ligand interaction on receptor expression in purified NK cells

**Figure 5:**
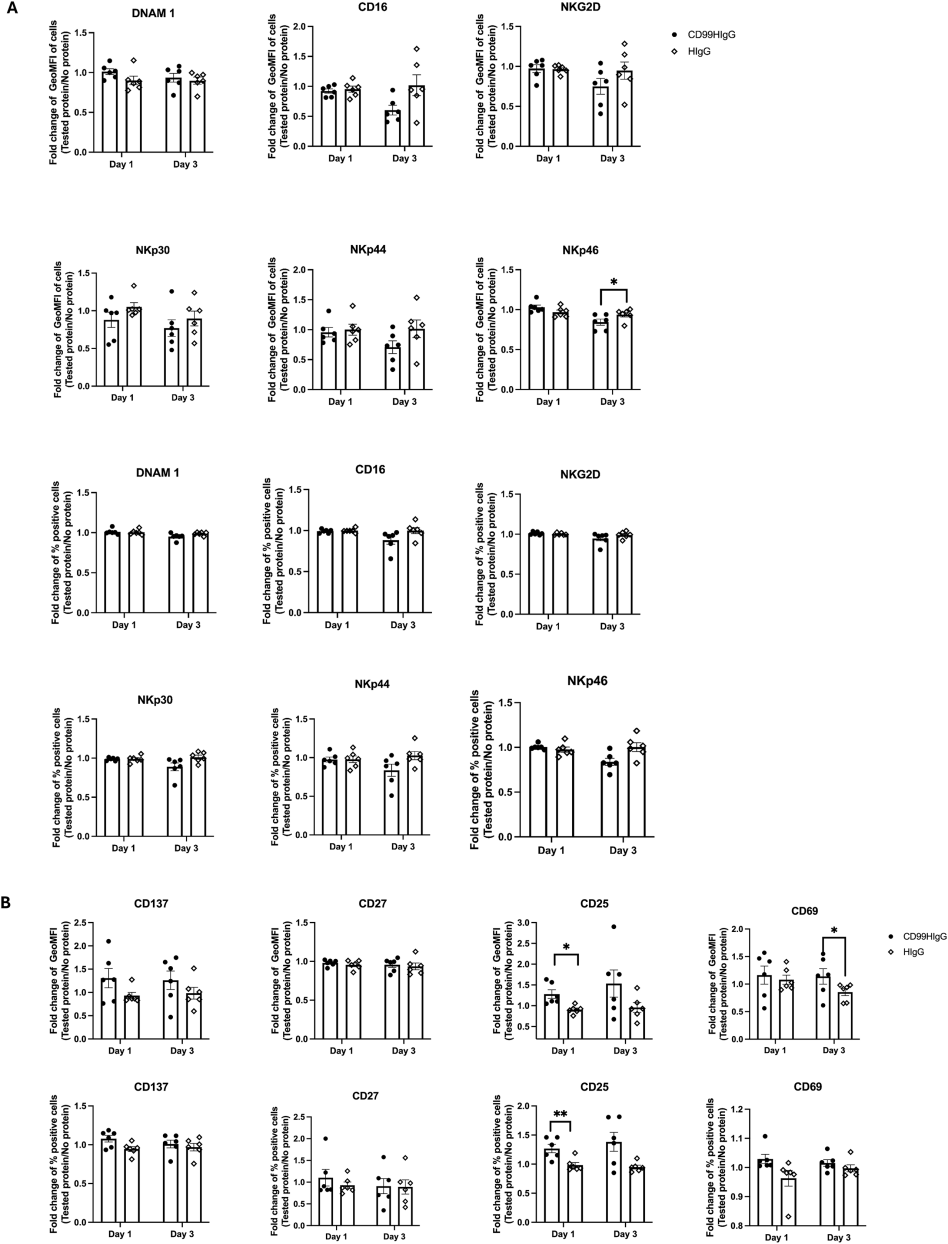
CD99HIgG selectively modulates NKp46, CD25, and CD69 expression in purified NK cells. Purified human NK cells were stimulated with IL-2 in the presence of CD99HIgG or control HIgG and analyzed on D1 and D3 by flow cytometry. **(A)** Fold changes in GeoMFI and percentage of activating receptors and NCRs on purified NK cells. **(B)** Fold changes in GeoMFI and percentage of activation-associated and co-stimulatory receptors on purified NK cells. Bar graphs presented as mean ± SD (n=6). Statistical analysis was performed using a paired t-test assuming normal distribution. *p < 0.05, **p < 0.01.

Purified NK cells were used to verify the modulatory effect of CD99HIgG on receptor expression. In the presence of CD99HIgG, the GeoMFI of NKp46 was significantly reduced on D3 compared with the control HIgG (p = 0.0275), whereas the percentage of NKp46^+^ NK cells showed a downward trend but not statistically significant (**Figure 5A**). In contrast, both GeoMFI and percentage of NK cells expressing CD25 were significantly increased following CD99HIgG treatment on D1 (p = 0.0309 and p = 0.0084). In addition, a significant increase of CD69 expression was also observed in D3 (p = 0.0282) (**Figure 5B**). No statistically significant differences were observed in the expression of other tested molecules. The representative FACS profiles showing GeoMFI of the tested molecules under different treatment conditions were presented in the **Supplementary Figure 2**.

### Functional assessment of CD99-mediated receptor modulation

Previous study demonstrated that CD99 regulates pro-inflammatory cytokine production under both unstimulated and OKT3-stimulated conditions (18). Since CD99 engagement under IL-2-stimulated conditions altered NK cell receptor expression in the present study, it was important to investigate whether these receptor changes were also associated with altered cytokine production in NK cells. Therefore, PBMCs were stimulated overnight with IL-2 and treated in the presence or absence of CD99HIgG. The intracellular cytokine staining was performed to examine the production of IL-6, TNF-α and IFN-γ in NK cells including monocytes and CD3^+^ cells. The gating strategy used to identify the different immune cell populations was shown in **Supplementary Figure 3**. As seen in **Supplementary Figures 4A**, **4B**, CD99HIgG treatment did not significantly affect cytokine production in NK cells. Similar results were obtained from CD3^+^ cells. However, in monocytes, there was significant upregulation of IL-6 and TNF-α expression, while the percentage of IL-6 positive cells showed a decreasing trend that did not reach statistical significance (**Supplementary Figure 4B**).

## Discussion

NK cells express several receptors on their surface. Their activation and effector functions are regulated by signals produced from activating and inhibitory receptors (1, 21, 22). The modulation of these receptor systems, therefore, represents a biologically significant indicator of altered NK activation dynamics. Accordingly, identifying novel pathways regulating NK cell receptor expression may help to elucidate mechanisms underlying NK cell activation and phenotypic diversity. CD99, a membrane protein, has been implicated in immune regulation and tumor microenvironment (23–25). Its ligands have been identified on NK cells, monocytes, and dendritic cells, and are distributed across different phenotypes of NK cells, with expression further upregulated following IL-2 stimulation (18, 19, 26). Notably, CD99 and its ligand interactions modulate inflammatory cytokines, while exerting limited effects on T cell proliferation or classical activation markers (18). These properties position CD99 as an immunomodulatory interface. Despite the reported involvement of CD99 in immune regulation, its effects on NK cell receptor modulation under stimulated conditions remain incompletely understood.

In the present study, we investigated the effects of CD99 and CD99 ligand interaction on NK cell activating receptor expression in different immune cell contexts under IL-2-stimulated conditions. We observed that the CD99-CD99 ligand interaction was associated with changes in NK cell receptor expression during IL-2-mediated activation. In PBMC culture, CD99HIgG exposure significantly reduced the expression of NKG2D and NKp30, while other NCRs (NKp44 and NKp46) also demonstrated a decreasing trend. In addition, a significant reduction in the percentage of NKG2D positive NK cells was observed, further supporting the modulatory effect of CD99 and CD99 ligand interaction on activating receptor profiles. NK cells demonstrate notable plasticity and can dynamically modify their receptor repertoires in response to cytokine exposure and intercellular interactions, thereby transitioning between effector, regulatory, and adaptive states (5, 9, 10). Of particular interest, NKp30 expression exhibits sensitivity to IL-2 availability, with cytokine deprivation selectively decreasing NKp30 surface expression and functional responsiveness (5, 27, 28). Therefore, the observed decrease in NKp30 and NKG2D expressions may reflect CD99-mediated modulation of NK cell activation status during prolonged IL-2 stimulation rather than direct suppression. This interpretation is supported by our previous finding demonstrating increased expression of CD99 ligands following immune cell activation (19), suggesting a potential role for CD99 ligands in regulating immune cell activity after activation.

Interestingly, differential responses were observed between PBMCs and purified NK cell cultures. In purified NK cells, NKp46 expression was significantly reduced, while other NCRs (NKp30 and NKp44) showed a downward trend, without reaching statistical significance. Despite the reduction in cytotoxic receptor expression, exposure to CD99HIgG resulted in a significant increase of CD69 and CD25 after IL-2 stimulation. CD25 (IL-2Rα) facilitates the assembly of the high affinity IL-2 receptor complex and promotes proliferation, and metabolic activation (10, 29). Clinical and experimental studies have demonstrated that redistribution of IL-2 bioavailability preferentially expands CD56 Bright NK subsets and enhances cytokine responsiveness, even when T-cell activation is limited (10). The concurrent upregulation of CD25 alongside diminished NCR expression may indicate a cytokine-responsive activation state accompanied by receptor adaptation.

PBMCs comprise heterogeneous immune cell populations, including T cells, B cells, NK cells, monocytes, and dendritic cells. Consequently, NK cell activation within PBMC cultures is influenced not only by direct stimulation but also by intercellular interactions and cytokine availability. Regulatory T cells can restrict IL-2 bioavailability, thereby limiting NK cell activation and responsiveness (10, 29). In addition, CD99 ligands are expressed not only on NK cells but also on monocytes and dendritic cells (18, 19). Therefore, within PBMC cultures, CD99HIgG may simultaneously interact with multiple immune cell subsets, facilitating both homotypic and heterotypic signaling interactions. These complex cellular interactions may contribute to the broader receptor modulation observed in PBMC cultures. In contrast, purified NK cells lack accessory immune cell population, resulting in a more simplified cellular environment. Under these conditions, receptor modulation appeared more selective and primarily characterized by significantly reduced NKp46 expression accompanied by increased CD25 and CD69 expression. These findings indicate that the effects associated with CD99-CD99 ligand interaction are influenced by the surrounding immune cell environment.

To further evaluate the functional relevance of receptor modulation, cytokine production was additionally assessed. No significant changes in IFN-γ, TNF-α and IL-6 production were observed in NK cells, suggesting that CD99-CD99 ligand interaction primarily affected by receptor expression rather than inducing strong cytokine responses under the conditions examined in this study. In PBMC cultures, increased IL-6 and TNF-α production was observed in monocytes, indicating that CD99-CD99 ligand interaction may also influence immune responses within mixed cell populations.

In summary, CD99-CD99 ligand engagement altered NK cell receptor profiles in both PBMCs and purified NK cell cultures. To our knowledge, this is the first study to demonstrate the engagement of CD99 and its ligands alters the expression of multiple activating receptors on NK cells and exhibits differential effects between PBMCs and purified NK cell systems. These findings contribute to the current understanding of NK cell biology by identifying CD99-CD99 ligand interaction as a potential regulator of NK cell receptor modulation under activated conditions, with possible implications for immune cell regulation and NK cell-based immunotherapy. Further studies investigating cytotoxicity and degranulation responses may help clarify the broader functional implications of CD99-CD99 ligand interaction in NK cell biology.

## Data Availability

The original contributions presented in the study are included in the article/**Supplementary Material**. Further inquiries can be directed to the corresponding author.
