# Supplementary material for: Interaction of CD99 and its ligands regulates immunoregulatory pathways in NK cells
Source: Front Immunol. 2026 Jun 4;17:1840402. doi: 10.3389/fimmu.2026.1840402 (PMC13275423; doi:10.3389/fimmu.2026.1840402)
Supplement: Supplementary file 1 [file DataSheet1.docx]

Supplementary Material


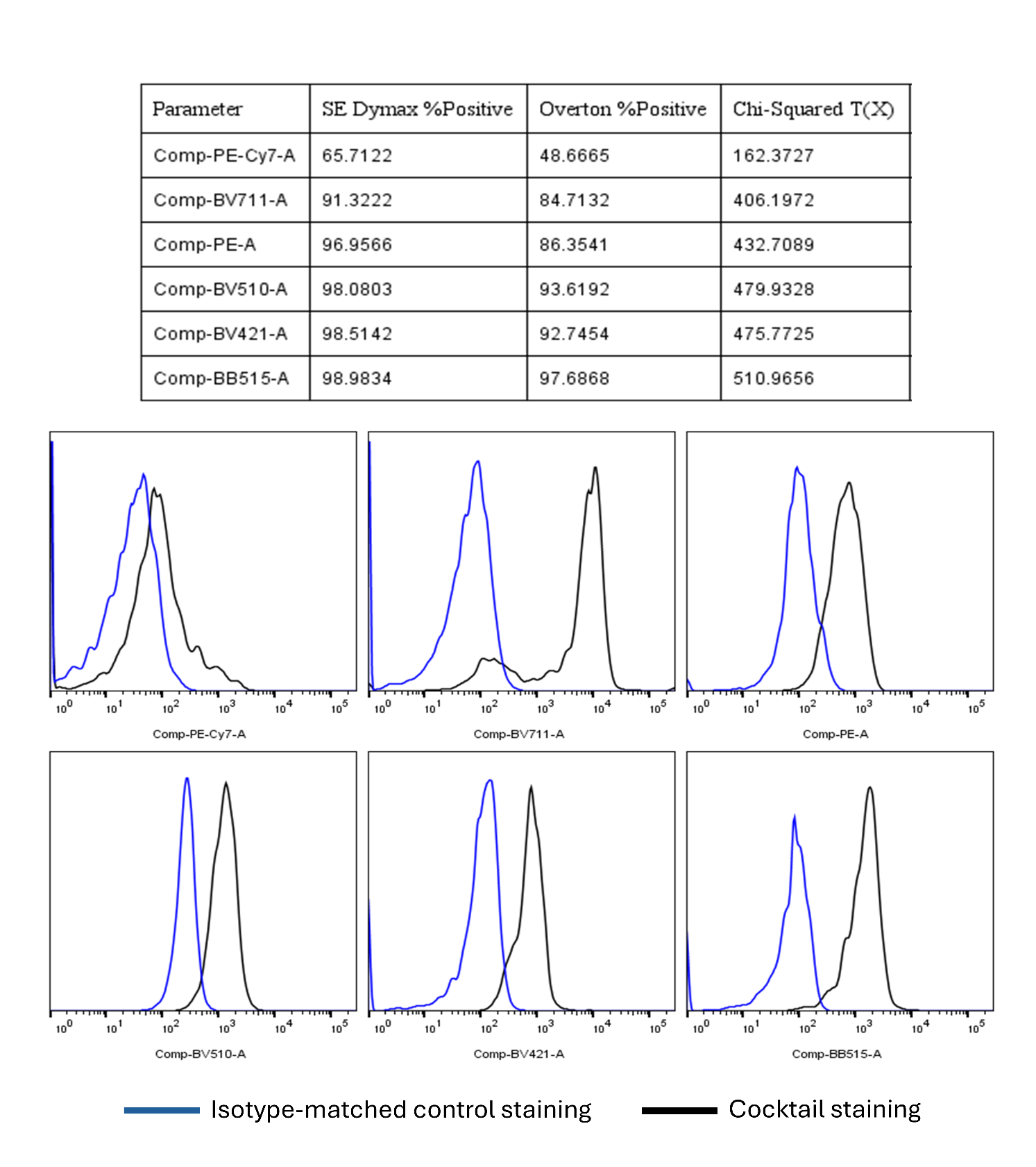
Supplementary Figure 1. Representative analysis of marker positivity using the Comparison Population tool. Marker expression from cocktail staining (black color) was compared with the corresponding isotype-matched control staining (blue color) for each tested marker under the same treatment condition. Overton % Positive values were subsequently used for further statistics analysis.


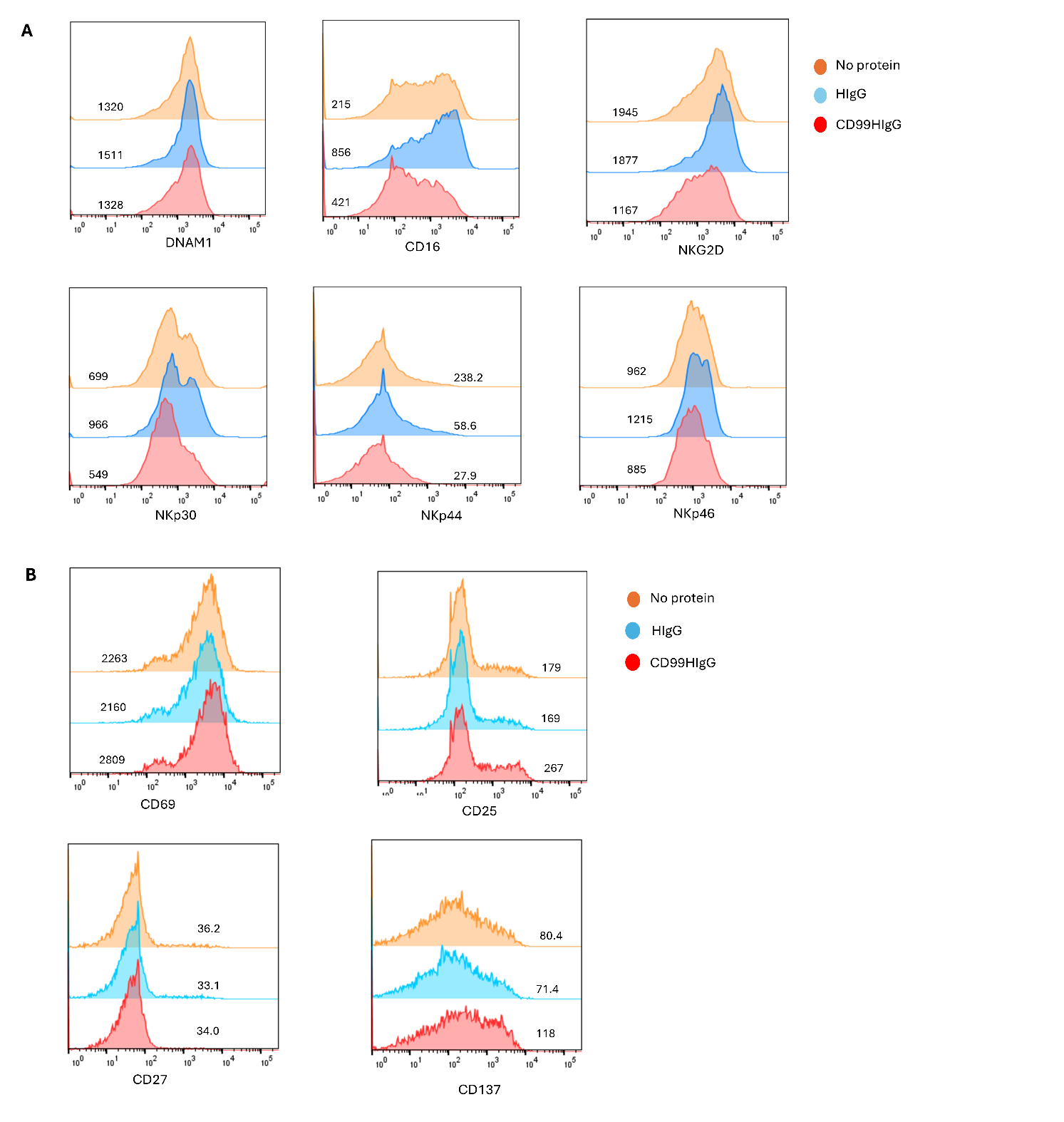


**Supplementary Figure 2.** Representative flow cytometry histograms at D3 after stimulation showing the GeoMFI of activating receptors and NCRs (A), and co-stimulatory receptors (B) expressed on purified NK cells under different treatment conditions (CD99HIgG, HIgG, and no protein).


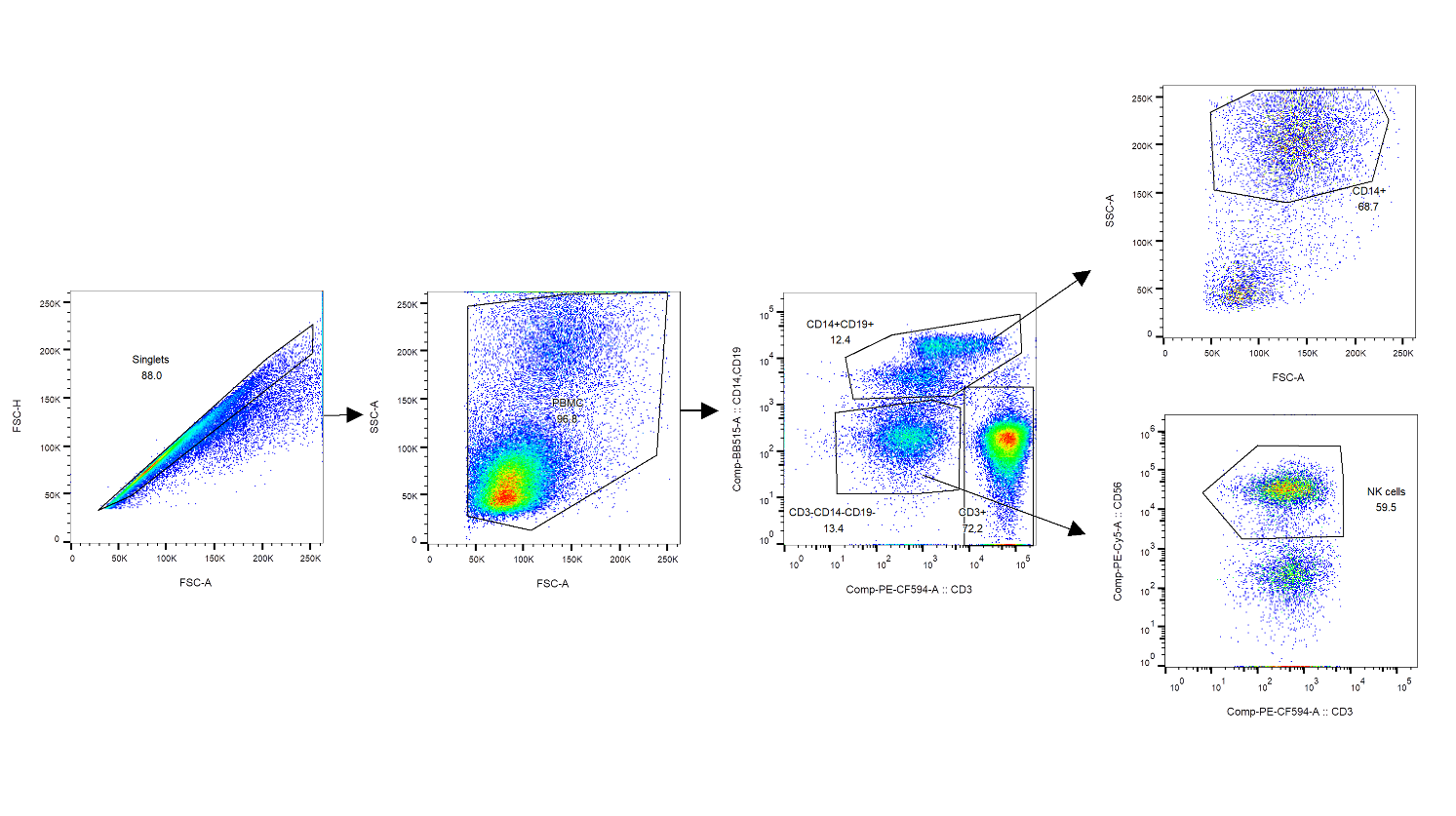


**Supplementary Figure 3. Gating strategy used to identify different immune cell popoulations.** Singlets were initially gated using FSC-A and FSC-H, followed by selection of PBMC populations based on FSC-A and SSC-A. The gated PBMCs were then analyzed using CD3-PECF594 together with CD14/ CD19-FITC staining to identify CD3^+^ and CD3^-^ populations. CD3^-^CD14^-^ CD19^-^ and CD3^-^CD14^+^CD19^+^ populations were subsequently gated. The CD3^-^CD14^-^ CD19^-^ population was further analyzed using CD3-PECF594 and CD56-PECy5 to identify NK cells, whereas the CD3^-^CD14^+^CD19^+^ population was plotted based on FSC-A and SSC-A characteristics to identify monocytes. Cytokine expression in each population was evaluated by GeoMFI and percentage of cytokine positive cells using Comparison Population tool.


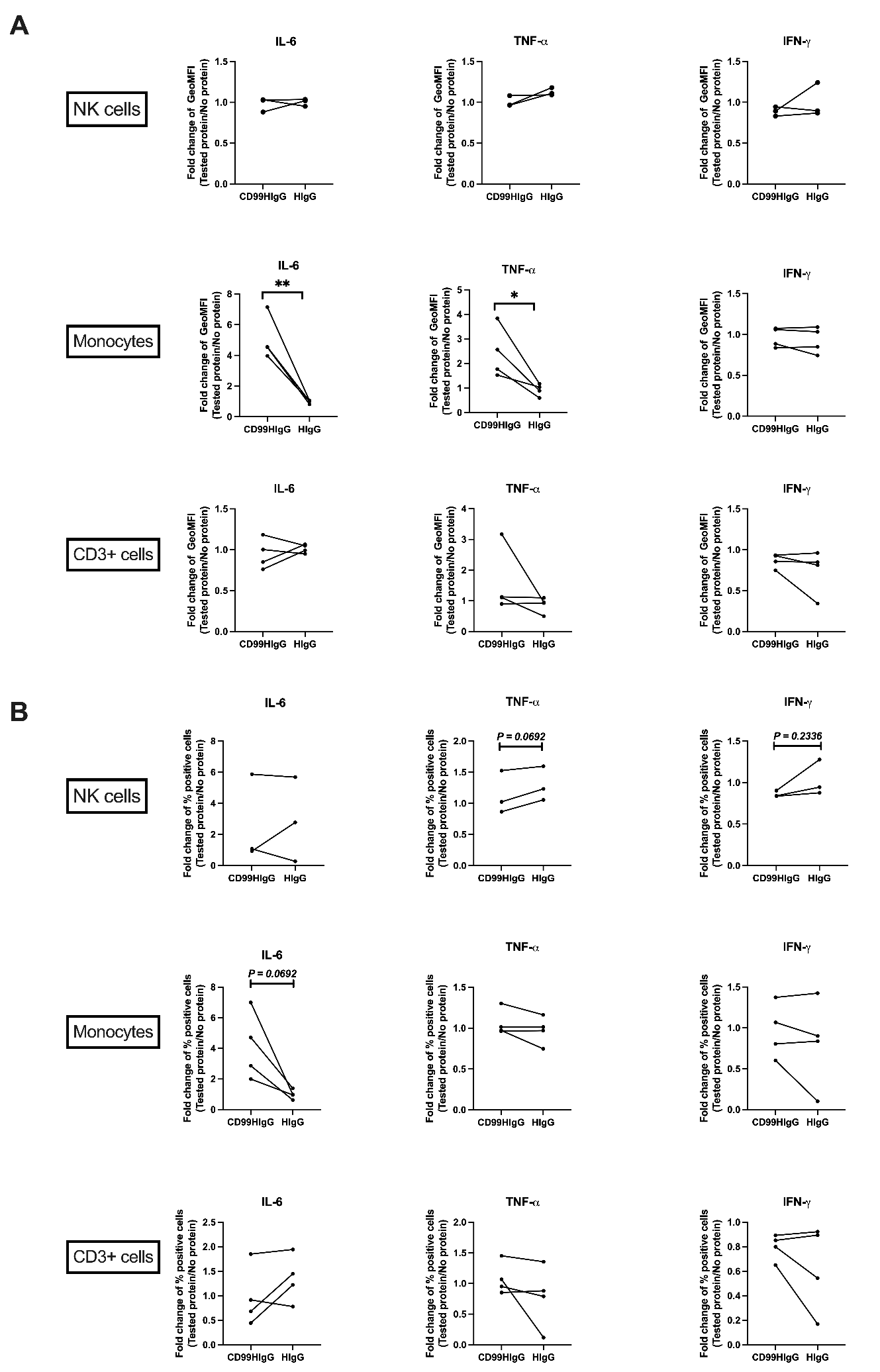


**Supplementary Figure 4. Functional assessment of CD99-mediated receptor modulation through intracellular cytokine analysis. (A)** Fold changes in GeoMFI of cytokines (IL-6, TNF-α and IFN-γ) in NK cells, monocytes and CD3^+^ cells under different conditions (CD99HIgG and control HIgG) following IL-2 stimulation. (B) Fold changes in percentage of cytokine positive NK cells, monocytes and CD3^+^ cells under different treatment conditions (n=3 for NK cells; n=4 for monocytes and CD3^+^ cells). Each dot represents an individual donor, and horizontal lines connect paired samples from the same donor. Statistical analysis was performed using a paired t-test assuming normal distribution. ** p < 0.05, ** p < 0.01*.
